# Some of the Clinical Aspects of Pneumonia

**Published:** 1906-01-13

**Authors:** 


### SOME OF THE CLINICAL ASPECTS OF PNEUMONIA.

The following notes are taken from a clinical lecture by Dr. Donald W. C. Hood.[1] The primary symptom of an attack of pneumonia, when the disease commences in the lower lobe, may be pain, often very severe pain, referred entirely to the abdomen. Unless this is borne in mind the true diagnosis may be altogether overlooked, and the case regarded as one of some form of acute abdominal disease; this is all the more likely to occur when, as sometimes happens, there is vomiting or other suggestion of gastric irritation. In such cases, at the outset, there are no physical signs of pulmonary disease, and it may be impossible to advance a confident diagnosis of the cause of the pain. Still there are certain facts which may be of value, and may prevent a mistaken conclusion that the disease is essentially sub-diaphragmatic. First among these is the rate of the respiration. When the respiration rate numbers 30 to 40 per minute, and there is with it a quick, sharp pulse and a temperature of 102°, the probability of some acute intra-thoracic disease is great, and this is increased rather than lessened by the presence of severe abdominal pain, associated with distinct hyperæsthesia of the skin. No doubt some rapidity of respiration may accompany the onset of abdominal inflammations, but here the temperature and pulse are usually lower than in the early stage of pneumonia. The practical point to remember is that severe abdominal pain, sometimes accompanied by vomiting, may find its interpretation in acute pulmonary disease, the usual physical attendants of which may be postponed for several days. This is all the more important in view of the growing disposition to open the abdomen for exploratory purposes in cases of more or less uncertain diagnosis.

Pneumonia, the result of the poison of influenza, has a much more anxious prognosis than the usual form of the disease. It is characterised by extreme asthenia, and often by copious perspiration; the range of temperature is not uniformly so high, and is far more irregular. From the rapid changes which occur in the physical signs in influenzal pneumonia it may be conjectured that the pathological condition in the lungs is one of rapid engorgement or œdema.

The "crisis" is a prominent fact in the natural history of pneumonia. The date is postponed in direct relationship to the amount of lung tissue involved in the pathological process. Thus it occurs earlier with a limited than with an extensive lesion. The position is always an anxious one when the appearance of the crisis is delayed. The following may be mentioned as circumstances which interfere with a typical defervescence. Pericarditis, pleurisy, more especially of the purulent variety, the occurrence of pneumonia in the opposite lung, and bronchitis or catarrhal inflammation is not uncommon in children. In some cases influenzal pneumonia has a typical crisis, whilst in others no such event can be recognised. Particularly is it necessary to remember in cases of pneumonia where the temperature does not fall the possibility of empyema. This complication often develops so insidiously and without any event to mark its occurrence that it may readily be overlooked, and this is the more likely to occur, as the changes in the breath sounds and other evidences which suggest consolidation may remain without substantial modification, even when the fluid accumulation within the pleural cavity is considerable in amount. One fact of significance in connection with these residual effusions is the presence of a cough which is essentially paroxysmal in character, comes on after movement, and is unattended by any expectoration. Such a cough following on the ordinary symptoms of acute pneumonia may be regarded as pathognomonic of the presence of fluid, though the volume of fluid may be very small.

Another practical point in connection with pleural effusion when this occurs in the left side is that occasionally it is found that the heart is not displaced. This has been shown to be due to adhesions, the results of a former pleurisy.

[1] The Lancet, December 30, 1905.

